# Structural analysis of *Clostridium acetobutylicum* ATCC 824 glycoside hydrolase from CAZy family GH105

**DOI:** 10.1107/S2053230X15012121

**Published:** 2015-07-29

**Authors:** Katherine L. Germane, Matthew D. Servinsky, Elliot S. Gerlach, Christian J. Sund, Margaret M. Hurley

**Affiliations:** a Oak Ridge Associated Universities, 4692 Millennium Drive, Suite 101, Belcamp, MD 21017, USA; b RDRL-SEE-B, US Army Research Laboratory, 2800 Powder Mill Road, Adelphi, MD 20783, USA; c Federal Staffing Resources, 2200 Somerville Road, Annapolis, MD 21401, USA; d RDRL-SEE-B, US Army Research Laboratory, 4600 Deer Creek Loop, Aberdeen Proving Ground, MD 21005, USA

**Keywords:** *Clostridium acetobutylicum*, pectin, unsaturated rhamnogalacturonyl hydrolase, glycoside hydrolase, GH105

## Abstract

*Clostridium acetobutylicum* ATCC 824 gene CA_C0359 encodes a putative unsaturated rhamnogalacturonyl hydrolase (URH) with distant amino-acid sequence homology to YteR of *Bacillus subtilis* strain 168. YteR, like other URHs, has core structural homology to unsaturated glucuronyl hydrolases, but hydrolyzes the unsaturated disaccharide derivative of rhamnogalacturonan I. The crystal structure of the recombinant CA_C0359 protein was solved to 1.6 Å resolution by molecular replacement using the phase information of the previously reported structure of YteR (PDB entry 1nc5) from *Bacillus subtilis* strain 168. The YteR-like protein is a six-α-hairpin barrel with two β-sheet strands and a small helix overlaying the end of the hairpins next to the active site. The protein has low primary protein sequence identity to YteR but is structurally similar. The two tertiary structures align with a root-mean-square deviation of 1.4 Å and contain a highly conserved active pocket. There is a conserved aspartic acid residue in both structures, which has been shown to be important for hydration of the C=C bond during the release of unsaturated galacturonic acid by YteR. A surface electrostatic potential comparison of CA_C0359 and proteins from CAZy families GH88 and GH105 reveals the make-up of the active site to be a combination of the unsaturated rhamnogalacturonyl hydrolase and the unsaturated glucuronyl hydrolase from *Bacillus subtilis* strain 168. Structural and electrostatic comparisons suggests that the protein may have a slightly different substrate specificity from that of YteR.

## Abstract 2

The crystal structure of the protein product of the *C. acetobutylicum* ATCC 824 gene CA_C0359 is structurally similar to YteR, an unsaturated rhamnogalacturonyl hydrolase from *B. subtilis* strain 168. Substrate modeling and electrostatic studies of the active site of the structure of CA_C0359 suggests that the protein can now be considered to be part of CAZy glycoside hydrolase family 105.

## 1. Introduction   

*Clostridium acetobutylicum*, a bacterium used to produce acetone, butanol and ethanol from the fermentation of carbohydrates, is capable of using pectin as a feedstock (Schink *et al.*, 1981 ▸). Little is known about pectin degradation by *C. acetobutylicum*; however, the degradation of the polygalacturonan (PGA) and rhamnogalacturonan-I (RG-I) backbones of pectin by plant pathogens has been thoroughly studied, and many of the known pectinolytic enzymes share distant homology to putative gene products encoded by the *C. acetobutylicum* genome (Collmer & Keen, 1986 ▸; Prade *et al.*, 1999 ▸; Ochiai *et al.*, 2007 ▸; Marín-Rodríguez *et al.*, 2002 ▸; Nolling *et al.*, 2001 ▸).

**Figure 1:**
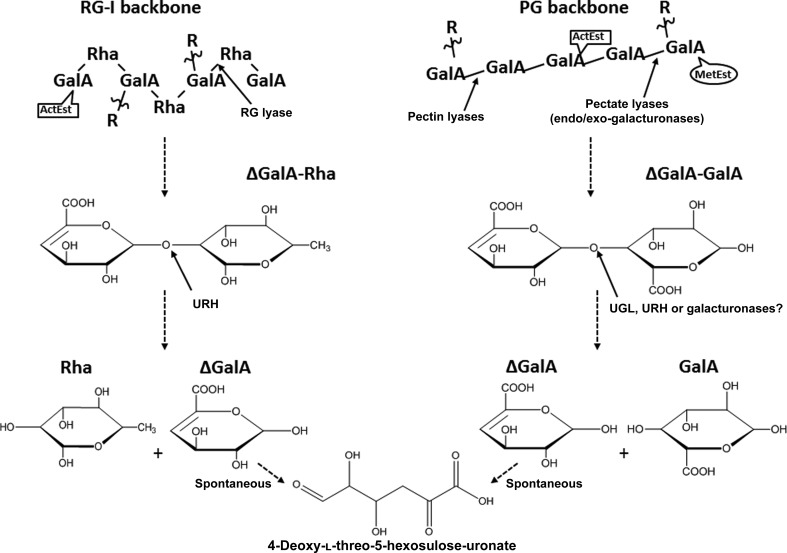
General degradation pathway of the pectin backbones of polygalacturonan (PGA) and rhamnogalacturonan-I (RG-I) by bacterial enzymes to obtain monomers for metabolic processes. The PGA backbone is targeted at the 1–4 α-linkage between galacturonic acids (GalA) by pectate lyases and pectin lyases, which cleave the backbone until single and small polysaccharides remain. The reaction will leave a saturated and unsaturated GalA. The small polysaccharides containing terminal GalA are imported into the bacterium and then targeted by either UGL, URH or galacturonases to leave a saturated GalA and unsaturated GalA. The RG-I backbone is degraded in a similar fashion by RG lyases, leaving a saturated rhamnose (Rha) and a polysaccharide containing an unsaturated terminal GalA. The short-chain polysaccharides follow the same process as PGA polysaccharides, and once inside the cell are cleaved by URH, leaving a rhamnose and unsaturated GalA. In both instances the unsaturated GalA may spontaneously open to 4-­deoxy-l-threo-5-hexosulose-uronate. *R*– indicates polysaccharide side chains linked to both backbones. The backbones are also decorated with acetyl esters (ActEst) and methyl esters (MetEst).

Fig. 1 ▸ shows a simple schematic of the HGA and RG-I degradation process. Pectate lyases and pectin lyases cleave between the galacturonate residues of PGA *via* β-elimination, leaving a terminal unsaturated galacturonate (ΔGalA), whereas endo-galacturonases and exo-galacturonases cleave the glycosidic linkage through hydrolysis (Markovic & Janecek, 2001 ▸; Giovannoni *et al.*, 1989 ▸; Prade *et al.*, 1999 ▸). Rhamnogalacturonan lyase cleaves between rhamnose (Rha) and galacturonate of RG-I, creating an ΔGalA at the non­reducing end (Ochiai *et al.*, 2007 ▸; Laatu & Condemine, 2003 ▸). After importation, further cleavage of HGA and RG-I short-chain unsaturated polysaccharides by unsaturated glycoside hydrolases occurs.

Unsaturated glycoside hydrolases are intracellular proteins and can be divided into two carbohydrate-active enzyme (CAZy) families: GH105 and GH88. The core structure of GH105 hydrolases are closely related to those of GH88, although they differ in substrate specificity and sequence identity (Collén *et al.*, 2014 ▸; Itoh, Hashimoto *et al.*, 2006*b* ▸). Itoh and coworkers identified GH105 as a novel family of proteins that have a gate loop that occludes the active site of GH105 proteins and is not found in the GH88 proteins, novel substrate activity and low sequence similarities to GH88 proteins. The gate loop prevents the larger substrates of GH88 proteins from binding properly in the GH105 pocket, thereby imposing substrate specificity (Itoh, Ochiai *et al.*, 2006*b* ▸). The known substrates of the GH88 family are mammalian-derived, including unsaturated glucuronyl *N*-acetyl­galactosamine (ΔGlcA-GalA; Itoh, Hashimoto *et al.*, 2006*b* ▸; Maruyama *et al.*, 2009 ▸). GH105 proteins have been shown to cleave plant-based and algae-based substrates by hydrolyzing small two-chain and three-chain unsaturated polysaccharides containing an unsaturated uronyl saccharide at the non­reducing end (Yip & Withers, 2006 ▸; Garron & Cygler, 2010 ▸; Coutinho & Henrissat, 1999 ▸). The characterized members of the GH105 family mainly consist of unsaturated rhamno­galacturonyl hydrolases (URHs), which attack unsaturated rhamnogalacturonan from RG-I (Itoh, Ochiai *et al.*, 2006*a* ▸,*b* ▸). The substrate specificity of GH105 proteins correlates to residues found in the active site and the loop lengths surrounding the site (Collén *et al.*, 2014 ▸). The suggested mechanism for both families requires hydration at the C=C bond of the unsaturated sugar, resulting in glycosidic cleavage (Jongkees & Withers, 2011 ▸). The hydrolysis mechanism releases the unsaturated sugar, which in the instance of galacturonate can spontaneously convert to 4-deoxy-l-threo-5-hexosulose-uronate (Itoh, Hashimoto *et al.*, 2006*b* ▸).

Here, we begin to analyze the CA_C0359 gene product, annotated by the Kyoto Encyclopedia of Genes and Genomes (KEGG) as a URH (GH105; Keis *et al.*, 2001 ▸; Kanehisa & Goto, 2000 ▸; Coutinho & Henrissat, 1999 ▸). Transcriptional studies and structural analysis of the CA_C0359-derived protein product presented here support its annotation as a GH105 family protein with an active pocket capable of binding unsaturated polysaccharides containing an unsaturated galacturonate. We identify CA_C0359 as a GH105 unsaturated glycoside hydrolase based on a structural analysis, with the potential to cleave the substrates of its homolog YteR and possibly other substrates.

## 2. Materials and methods   

### 2.1. Bacterial growth   

*C. acetobutylicum* ATCC 824 was the subject strain for this experiment. Clostridial growth medium (CGM) was used for routine growth and cells were propagated in an anaerobic environment as described previously (Servinsky *et al.*, 2010 ▸). For gene-expression studies, the glucose in CGM was replaced by 0.5% arabinose, galacturonic acid, glucose, lactose, pectin or polygalacturonic acid. One Shot TOP10 (Life Technologies) and BL21 (DE3) (New England Biolabs) *E. coli* cells were used for molecular cloning and protein purification, respectively.

### 2.2. Quantitative PCR (qPCR)   

Cells from cultures grown to mid-exponential growth phase (OD_600_ of 0.45–0.56) were harvested as described previously (Supplementary Table S1; Servinsky *et al.*, 2010 ▸). PCR primers (Supplementary Table S2) were designed using *Primer*3 (Untergasser *et al.*, 2007 ▸) to generate ∼125 bp products, and primer pair efficiency was determined as described previously (Servinsky *et al.*, 2010 ▸).

### 2.3. Plasmid construction   

Gene CA_C0359 was PCR-amplified from *C. aceto­butylicum* ATCC 824 genomic DNA using primers 359NdeIF (GATCCATATGATGCAAAAATATTCTAAATTAATGGCAG) and 359XhoR (AAGTGTTTCGTATTCGTAAGATGCAAGTAAGCTCGAGGATC) to introduce restriction-enzyme sites for NdeI and XhoI at the putative transcriptional start site and the 3′ end of the gene, also removing the stop codon, respectively. The PCR product was cloned into pTXB1 using standard protocols (Maniatis *et al.*, 1982 ▸); the resulting plasmid was named p359-intein and was used to produce the CA_C0359 gene product with a C-terminal intein (chitin-binding domain) tag.

### 2.4. Expression and purification of p359-intein fusion protein   

Chemically competent *E. coli* BL21 (DE3) cells (New England Biolabs) were transformed with p359-intein using the manufacturer’s protocol. Cultures of transformed cells were grown to an OD_600_ of 0.6 in 1 l LB Lennox broth supplemented with 50 µg ml^−1^ ampicillin at 37°C in a shaking incubator and were then transferred to 21°C for 30 min in a shaking incubator. Expression and purification of CA_C0359 protein was performed using previously established protocols (Cantor & Chong, 2001 ▸; Chong *et al.*, 1997 ▸; IMPACT, New England Biolabs). The molecular mass of the CA_C0359 protein product is 41.7 kDa.

Partially purified protein was concentrated to 5 ml using an Amicon Ultra centrifugal device (MWCO 9K, Millipore) and dialyzed in a MWCO 9K dialysis cassette (Pierce) into 4 l S-75 buffer (150 m*M* NaCl, 15 m*M* Tris pH 7.5) for 24 h at 4°C. The dialyzed protein sample was further purified on a Superdex 75 size-exclusion column (GE Healthcare) in S-75 buffer. The fractions containing the CA_C0359 protein, as determined by SDS–PAGE, were concentrated *via* an Amicon Ultra centrifugal device (MWCO 9K, Millipore).

### 2.5. Crystallization and data collection   

Native crystals were obtained by mixing 2 µl protein sample (7.5 mg ml^−1^) with 2 µl reservoir solution consisting of 0.1 *M* Tris pH 7.75, 16%(*w*/*v*) polyethylene glycol 4000 (Hampton Research) at 21°C using the hanging-drop vapor-diffusion method. Crystals were cryoprotected by soaking in 20% glycerol in reservoir solution, which was followed by liquid-nitrogen flash-cooling. Diffraction data were collected on the NSLS X29a beamline at Brookhaven National Laboratory using an ADSC Quantum 315 CCD detector at a wavelength of 1.075 Å.

### 2.6. Structure determination and refinement   

The structure was solved *via* the molecular-replacement method using *MOLREP* (Vagin & Teplyakov, 2010 ▸) with the structure coordinates of YteR from *B. subtilis* strain 168 (PDB entry 1nc5; Zhang *et al.*, 2005 ▸) as a model. The model was refined using *REFMAC*5 (Murshudov *et al.*, 2011 ▸) and multiple iterations of *phenix.refine* (Afonine *et al.*, 2012 ▸), with manual rebuilding performed in *Coot* (Emsley *et al.*, 2010 ▸). Solvent molecules were initially introduced with *ARP*/*wARP* (Cohen *et al.*, 2008 ▸) and subsequently refined in *phenix.refine* (Afonine *et al.*, 2012 ▸). Results were checked with *MolProbity* (Chen *et al.*, 2010 ▸) within *PHENIX* (Adams *et al.*, 2010 ▸).

### 2.7. Substrate-structure modeling   

Carbohydrate ligands were constructed with the *GLYCAM* carbohydrate builder (http://www.glycam.org). Docking was performed using the *SwissDock* web service (Grosdidier *et al.*, 2011*a* ▸,*b* ▸) and the *PATCHDOCK* web service (Duhovny *et al.*, 2002 ▸; Schneidman-Duhovny *et al.*, 2005 ▸).

### 2.8. Figure preparation   

The sequence alignment was prepared using *ESPript*3.0 (Robert & Gouet, 2014 ▸). Images were prepared with *VMD* (Humphrey *et al.*, 1996 ▸) and *Chimera* (Pettersen *et al.*, 2004 ▸). The electrostatic surface potential was calculated using the *PDB*2*PQR* server (Dolinsky *et al.*, 2004 ▸, 2007 ▸) and *APBS* (Baker *et al.*, 2001 ▸).

## 3. Results and discussion   

### 3.1. Identification of a potential GH105 family protein   

Examination of the CAZy database identified eight genes with possible pectin-backbone degradation activity in *C. acetobutylicum* ATCC 824. CA_C0359 was the only gene identified coding for a putative unsaturated glycoside hydrolase from CAZy family GH105, and did not contain a predicted Gram-positive export signal. Transcriptional analysis using quantitative PCR indicated CA_C0359 mRNA was induced threefold and sixfold during growth on pectin and polygalacturonic acid, respectively, when compared with growth on glucose (Supplementary Fig. S1). Growth on galacturonic acid monomers did not induce CA_C0359, indicating that a degradation product of pectin or PGA is required for gene induction. Additionally, a lack of induction during growth on galacturonic acid, arabinose or lactose indicates that CA_0359 is not subject to catabolite repression.

Sugar-specific induction of genes reveals information about the function of the expressed enzymes and their roles in a metabolic pathway (Martens *et al.*, 2011 ▸; Servinsky *et al.*, 2010 ▸). Induction of CA_C0359 mRNA by PGA suggests that the annotation of the gene as a URH was incorrect. The PGA backbone is a major component of pectin (Prade *et al.*, 1999 ▸), and thus induction of CA_C0359 on this substrate suggests that the protein might target a PGA-derived substrate. *C. aceto­butylicum* is capable of subsisting on galacturonic acid monomers and therefore may not have a strict requirement to process polysaccharides intracellularly (Servinsky *et al.*, 2014 ▸). In this instance, CA_C0359 would serve a redundant role to increase the speed of PGA metabolism. Alternatively, owing to the abundance of PGA in pectin, cells may use PGA as a pectin indicator, which could account for the increased induction of CA_C0359.

A domain enhanced lookup time accelerated *BLAST* (*DELTA-BLAST*; Boratyn *et al.*, 2012 ▸) search of the Protein Data Bank, which uses position-specific scoring matrices and has an improved performance in identifying distant homologs, was performed on CA_C0359. The highest scoring protein with a known function was the *B. subtilis* strain 168 protein YteR, with 38% identity. Itoh and coworkers have solved the crystal structures of the distant homologs YteR and YesR, both from *B. subtilis* strain 168. Both YteR and YesR have α-galacturonyl hydrolase activity and use unsaturated rhamnogalacturonan as a substrate. Neither use the ΔGlcA-GalNAc utilized by *B. subtilis* UGL from GH88. YesR and other proteins with known GH105 activity, including RhiN from *Erwinia chrysanthemi* and *Nu*_GH105 from *N. ulvanivorans*, have lower sequence similarity to CA_C0359 (Collén *et al.*, 2014 ▸; Hugouvieux-Cotte-Pattat, 2004 ▸). *Nu*_GH105 is structurally similar to YteR and YesR, possessing activity on a range of different oligosaccharides from algae, all of which had a sulfated rhamnose next to the unsaturated glycuronic acid residue at the nonreducing end (Collén *et al.*, 2014 ▸). RhiN also has activity on unsaturated rhamnogalacturonan, although its structure has not been determined. UGLs from GH88 have a low sequence identity in the same range as RhiN and YesR.

An *ESPript*3.0 (Robert & Gouet, 2014 ▸) multiple sequence alignment of the CA_C0359 amino-acid sequence with those of YteR, YesR, *Nu*_GH105, RhiN and a UGL from *B. subtillis* indicated there was some conservation among residues. However, UGL did not align well with the proteins as a group, despite the similarity in core structure and active residues between UGL and YteR (data not shown). When UGL was removed, several solvent-accessible residues in the active pockets were found to be conserved among the five proteins (Supplementary Fig. S2*a*). The conserved residues represented by YteR are Tyr41, Asp88, His132, Trp141, Asp143, Met147, His189, Trp211 and Trp217. These have all been structurally and experimentally shown to be involved in catalytic activity (Itoh, Ochiai *et al.*, 2006*a* ▸). Residues Tyr40 and Asp88 in YteR (residues 41 and 75 in CA_C0359) are a Trp and an Asn in RhiN and YesR, which Itoh and coworkers believe to be the basis behind the differences in optimal pH activity between YesR and YteR at pH 6 and pH 4 (Itoh, Ochiai *et al.*, 2006*b* ▸).

### 3.2. Crystal structure of CA_C0359   

**Figure 2:**
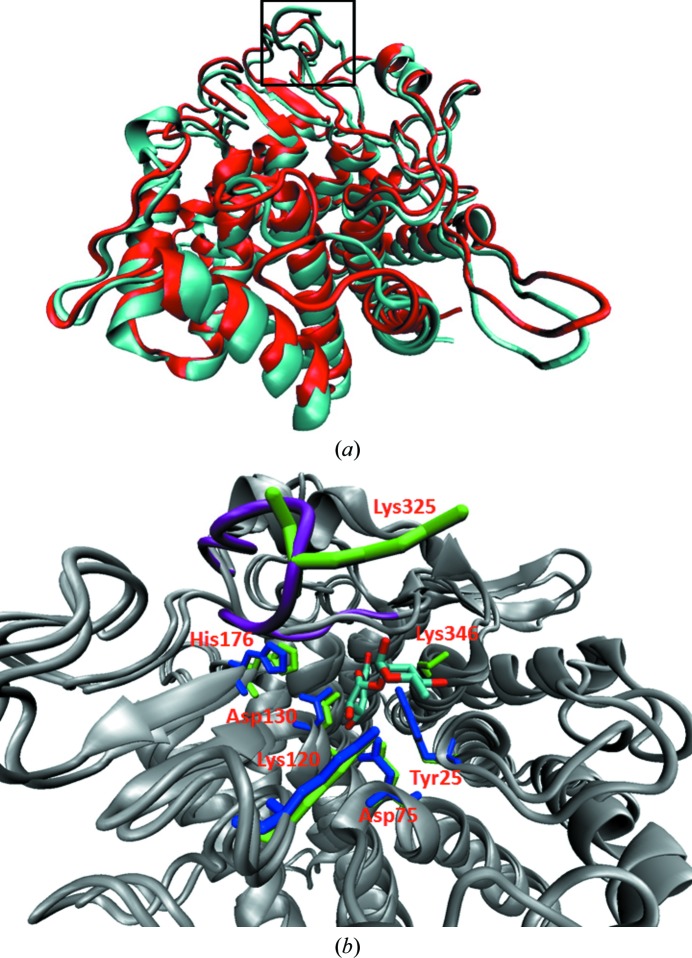
Structure alignment of the CA_C0359 protein with YteR (PDB entry 2gh4) and comparison of conserved active residues in the catalytic site. (*a*) CA_C0359 (cyan) aligned with the structure of YteR (red) with an r.m.s.d. of 1.4 Å; the protruding loop of CA_C0359 is boxed in black. (*b*) A comparison of active residue location of YteR with conserved residues of CA_C0359 on the protein alignment of CA_C0359 and YteR (gray), with the protruding loop of CA_C0359 colored magenta. Unsaturated rhamnogalacturonan, indicated by the red and cyan sticks, is bound to YteR (PDB entry 2gh4). The YteR residues highlighted in blue are Asp143, His189, Asp88, Tyr41 and Lys133. The conserved CA_C0359 residues labelled in red and highlighted in green include Asp130, His176, Asp75, Tyr25 and Lys120 and are seen to align well with the analogous YteR residues. Two additional lysines, Lys325 and Lys346, of CA_C0359 have been computationally determined to interact and coordinate substrate binding.

To yield further information on the possible activity of CA_C0359, the crystal structure of the protein was solved to 1.6 Å resolution (Table 1 ▸, Supplementary Fig. S3) and deposited in the Worldwide Protein Data Bank (PDB) with accession code 4wu0. Crystals of the CA_C0359 protein belonged to space group *P*2_1_2_1_2_1_, with two molecules in the asymmetric unit and a solvent content of approximately 47.6%, as determined using the Matthews coefficient (Matthews, 1968 ▸; Kantardjieff & Rupp, 2003 ▸). The structure was solved *via* molecular replacement using the phase information from YteR (PDB entry 1nc5; Zhang *et al.*, 2005 ▸). Structure refinement using the 1nc5 model resulted in a final *R* factor of 13.7% and an *R*_free_ of 16.3%. The CA_C0359 structure aligns with 1nc5 with a root-mean-square deviation of 1.4 Å (Fig. 2 ▸ *a*). The protein adopts a six-(α/α)-hairpin barrel fold with a small two-stranded β-sheet and helix overlaying the end of the barrel near the active pocket. The CA_C0359 structure closely adopts the fold of GH105 proteins based on a comparison made by Collén *et al.* (2014 ▸).

**Figure 3:**
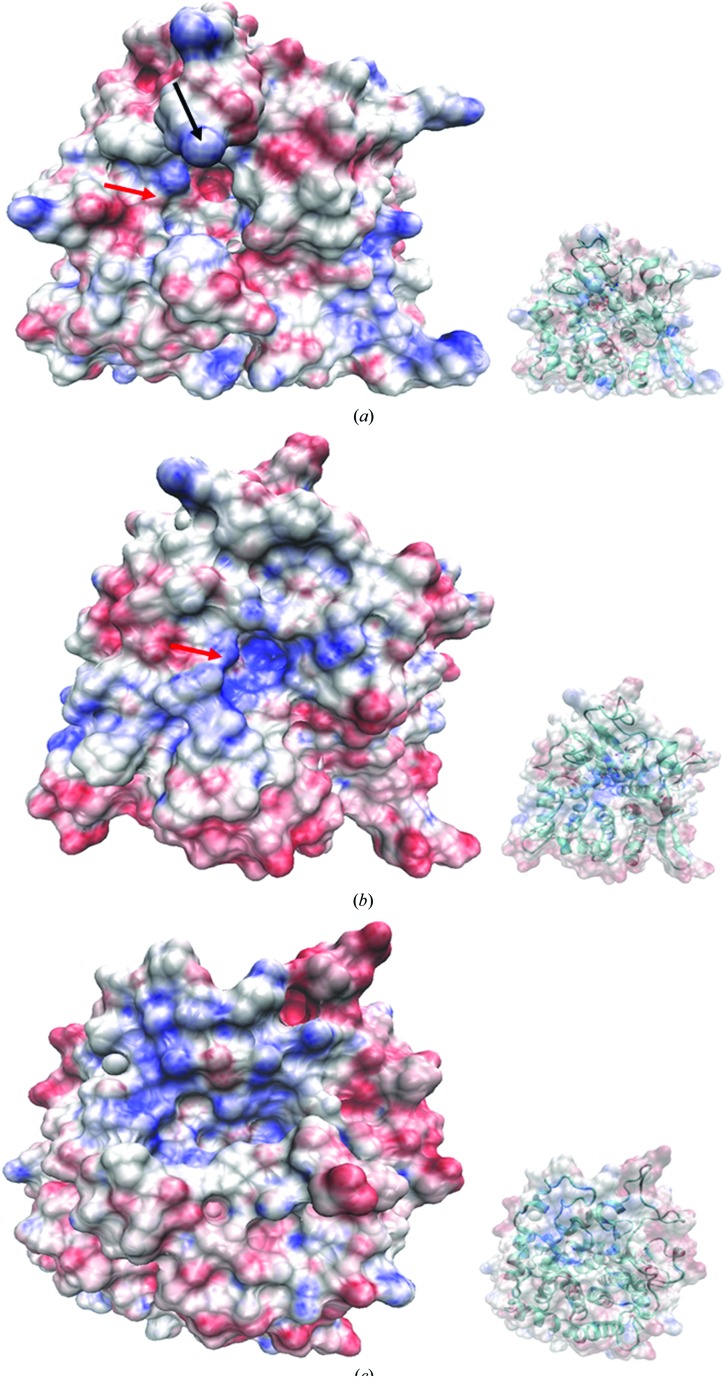
An electrostatic comparison of proteins from the GH105 and GH88 CAZy families. The surface electrostatic potentials of (*a*) CA_C0359, (*b*) YteR (PDB entry 2gh4) and (*c*) UGL from *B. subtilis* strain 168 (PDB entry 1vd5). The calculated surface electrostatic potentials are color-coded on surface renderings of the three crystal structures (red, negative; blue, positive). The structures are all oriented similarly to display electrostatics down into the active-site pocket. The extended loop of CA_C0359 is indicated by a black arrow. The gate loop in CA_C0359 and YteR is indicated by a red arrow. The smaller inset indicates the ribbon structure visible below the surface rendering to discern orientation details.

When the sequence alignment was fitted to a surface rendering of the CA_C0359 structure, the putative active-site pocket was highly concentrated with conserved residues (Supplementary Fig. S2*b*). Conserved residues included the highlighted residues from the active-site pocket of YteR (PDB entry 2d8l; Itoh, Ochiai *et al.*, 2006*b* ▸) seen in the sequence alignment and correspond to CA_C359 residues Tyr25, Asp75, His119, Trp128, Asp130, Met134, Arg200, His176, Trp198 and Trp204 (Fig. 3 ▸ *b*). Of these, Asp130, His176, Asp75 and Tyr25 are also considered to be functionally important for UGL (PDB entry 2fv1; Itoh, Hashimoto *et al.*, 2006*a* ▸) and correspond to Asp149, His193, Asp88 and Phe46. These are thought to be the active residues in the vinyl hydration of unsaturated sugars in YteR, UGL and likely CA_C0359 (Fig. 2 ▸ *b*). Analysis of sugar binding within the *B. subtilis* UGL structure stressed the important role of Trp42 in providing a stabilizing stacking interaction essential in binding the ΔGlcA of ΔGlcA-GalA (Itoh, Ochiai *et al.*, 2006*a* ▸). This interaction is not available in either the YteR or the CA_C0359 structures.

Itoh and coworkers postulated the effect of steric hindrance from a protruding loop (gate loop), also seen in CA_C0359, YesR and *Nu*_GH105, comprising residues 331–335 in YteR, which spans the top of the entrance to the active site and is not found in the UGL structure. ΔGlcA-GalNAc was able to fit into the YteR active pocket, but the ΔGlcA was rotated on its axis, thereby preventing hydrolysis. The ‘protruding loop’ 331–335 of YteR is replaced by a longer, more highly mobile loop (Fig. 2 ▸ *a*) comprising residues 320–330 in CA_C0359.

### 3.3. Surface electrostatic comparison   

To further investigate possible interactions between disaccharides and CA_C0359 within the context of UGL and YteR, an electrostatic analysis was performed. The active site of YteR is largely positive owing to the presence of surrounding histidines and arginines (Itoh, Ochiai *et al.*, 2006*a* ▸). This is suspected to play a role in concentrating the negatively charged end of the disaccharide. The ‘gating loop’ (residues 331–335) contributes to the ring of positive charge at the top of the active-site pocket, and may contribute to drawing sugars to the active site. Additional neutral patches exist near the top of the active-site pocket suitable for stabilizing the second (non-interacting) sugar ring. The active-site pocket of UGL is markedly more negative, and is less attractive to incoming negatively charged sugars despite more open access (Fig. 3 ▸ *c*). The character of the CA_C0359 active-site pocket is somewhere between that of YteR and UGL. The pocket surrounding the active site of CA_C0359 is lined with aromatic residues in a similar fashion to the UGL active site (Fig. 3 ▸ *a*; Itoh, Ochiai *et al.*, 2006*a* ▸). There are positive patches that provide attractive interactions at the top of and continuing down into the pocket (including the mobile loop 320–330). Similarly to YteR, there are additional surrounding patches of relatively neutral space suited for stabilizing the neutral (non-interacting) sugar ring.

### 3.4. Substrate modeling   

**Figure 4:**
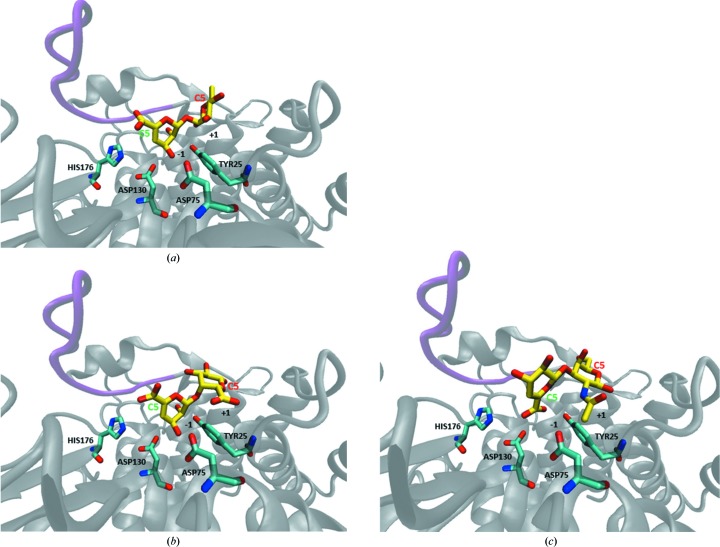
Substrates of both GH88 and GH105 computationally modeled into the active site of the CA_C0359 structure. The computationally predicted models (yellow) of (*a*) ΔGalA-Rha, (*b*) ΔGalA-GalA and (*c*) ΔGlcA-GalNAc bound to the ribbon model of the native CA_C0359 crystal structure. The known conserved residues Asp130, His176, Asp75 and Tyr25 are available for interaction with the carbohydrate and are explicitly shown in cyan. C5 of the reducing and nonreducing ends of the carbohydrate are labeled in red and green, respectively. H atoms are omitted from the visualization for clarity. The protein is shown in a gray cartoon representation, and the mobile loop (residues 320–330) is visible in the upper left portion of the figure and is colored magenta. Subsites for sugar binding are labeled −1 and +1.

ΔGlcA-GalNAc, ΔGalA-Rha and ΔGalA-GalA were docked into the active site of CA_C0359 using *SwissDock* and compared with the binding patterns found in the related PDB entries 4ce7 (Collén *et al.*, 2014 ▸), 2fv1 (Itoh, Hashimoto *et al.*, 2006*a* ▸), 2d8l (Itoh, Ochiai *et al.*, 2006*b* ▸) and 2gh4 (Itoh, Ochiai *et al.*, 2006*a* ▸). These disaccharides were chosen based on our qPCR studies and analysis performed by Itoh, Ochiai *et al.* (2006*b* ▸). Both the *PatchDock* and *SwissDock* docking web servers were used with default values, searching the entire protein with no input provided on potential binding sites or regions of interest. Both algorithms highlighted the region of protein incorporating the putative active pocket. This study represents an initial assessment of the CA_C0359 active pocket shape and gross electrostatic interactions, and their role in sugar binding, and does not portray the single ‘correct’ docked structure nor account for ligand flexibility (Nivedha *et al.*, 2013 ▸). All three disaccharides fit into the −1 to +1 subsites and are in reasonable proximity to known conserved catalytic residues without rotation of the sugar along the axis (Fig. 4 ▸). The sugar fit is noteworthy owing to the previously described rotation of ΔGlcA-GalNAc during binding to YteR (Supplementary Fig. S4). The representative *PatchDock* results demonstrate that the conserved residues Asp130, His176, Asp75 and Tyr25 are available for binding the three disaccharides of interest. While the mobile loop of CA_C0359 (residues 320–330) may provide some gating action, it is unlikely to completely block access to the active-site pocket. Some docking results demonstrated that some mobile loop residues (including Asp326) may furnish additional positive interactions with the sugar to facilitate attracting it to the active-site pocket. Other solvent-accessible residues with possible function during saccharide binding in the CA_C0359 structure include Lys120, Lys325 and Lys346.

Using the enzymatic reaction model of YteR with ΔGalA-Rha substrate of Itoh and coworkers, we hypothesize, based on conservation between active sites, that Asp130 of CA_C0359 will form a hydrogen bond to a water molecule that is stabilized by His176 (Itoh, Ochiai *et al.*, 2006*a* ▸,*b* ▸). The aspartic acid residue then donates a proton to the double bond at the C4 atom of ΔGalA, and less likely ΔGlcA, and deprotonates the water molecule through a general acid/base reaction. The C4=C5 bond then hydrolyzes to produce an unstable intermediate, which in turn isomerizes to the aldehyde 4-deoxy-l-threo-5-hexosulose-uronate. The CA_C0359 protein will hereafter be referred to as *Cac*_GH105 owing to the substrate source, sequence identity, structural analysis and conserved active-site configuration.

Substrate specificity in the GH105 family correlates with the loops surrounding the active site (Collén *et al.*, 2014 ▸). The active residues required for vinyl hydration and the gate loop are highly conserved among the GH105 family, including *Cac*_GH105. Other loops differ slightly in length and residue make-up, which affects electrostatic interactions, allowing room for different disaccharides and trisaccharides to bind in the subsites (Fig. 2 ▸ *a*, Supplementary Fig. S4). For example, the loops surrounding the active site of *Nu*_GH105 have a much more open configuration than in YteR, YesR and *Cac*_GH105 (Collén *et al.*, 2014 ▸). This allows tetrasaccharide-sized sugars containing unsaturated glucuronates and sulfated rhamnose at the nonreducing end to bind to the active site. The catalytic site of *Cac*_GH105 is slightly more open than those of YteR and YesR, and not as open as *Nu*_GH105, which may allow larger sugars to fit the +1 subsite, and possibly trioligosaccharides and tetraoligosaccharides. Conversely, the loop extending from the gate loop of *Cac*_GH105, which is not observed in the other GH105 structures, may play an important role in substrate specificity compared with other proteins in this family. The extended loop has high *B* factors and a charged lysine at the top of the loop (Fig. 2 ▸ *b*), which the docking study indicates may interact with bound oligosaccharide but does not affect the ability of the disaccharides listed above to fit into the active site. It is not clear whether this loop affects the size of the sugar or the length of the oligosaccharide that is able to bind to the active site. Taken with the electrostatic comparison and growth on pectin and PGA, *Cac*_GH105 may cleave a wider range of substrates than or a different range of substrates to YteR, including ΔGalA-Rha and ΔGalA-GalA, which may be decorated with side chains or be longer-chained oligosaccharides.

## Supplementary Material

PDB reference: *C. acetobutylicum* ATCC 824 glycoside hydrolase, 4wu0

Supporting Information.. DOI: 10.1107/S2053230X15012121/tb5084sup1.pdf

## Figures and Tables

**Table 1:** Crystallographic data-collection and refinement statistics for a native crystal of CA_C0359 (*Cac*_GH105) Values in parentheses are for the highest resolution shell.

Data collection	
	
Space group	*P*2_1_2_1_2_1_
*a*, *b*, *c* ()	53.3, 93.6, 156.7
Wavelength ()	1.075
Limiting resolution ()	19.91.60 (1.621.60)
Unique reflections	103657 (10151)
*R*_merge_ (%)†	8.6 (61.1)
Multiplicity	5.7 (3.2)
Completeness (%)	99.8 (98.8)
*I*/(*I*)	29.3 (4.5)
Refinement	
Resolution range ()	19.931.60 (1.661.60)
*R* factor (%)‡	13.7
*R*_free_ (%)	16.3
Non-H atoms	5872
Water molecules	835
R.m.s.d., bonds ()	0.009
R.m.s.d., angles ()	1.2
Average *B* factor (^2^)	
All atoms	20.4
Water molecules	32.8
Ramachandran plot	
Preferred (%)	99.2
Allowed (%)	0.8
Outliers (%)	0

† *R*_merge_ = 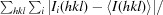 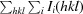 100.

‡ *R* factor = 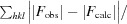 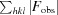 100.
