# Supplementary material for: Structural analysis of Clostridium acetobutylicum ATCC 824 glycoside hydrolase from CAZy family GH105
Source: Acta Crystallogr F Struct Biol Commun. 2015 Jul 29;71(Pt 8):1100–8. doi: 10.1107/S2053230X15012121 (PMC4528949; doi:10.1107/S2053230X15012121)
Supplement: Supplementary file 1 [file f-71-01100-sup1.pdf]

Acta Cryst F STRUCTURAL BIOLOGY COMMUNICATIONS

Volume 71 (2015)

Supporting information for article:

Structural analysis of *Clostridium acetobutylicum* ATCC 824 glycoside hydrolase from CAZy family GH105

Katherine L. Germane, Matthew D. Servinsky, Elliot S. Gerlach, Christian J. Sund and Margaret M. Hurley

Table S1 Culture OD at cell collection for transcriptional analysis by qPCR.

Replicate	Arab.	Gal.	Gluc.	Lact.	Pectin	Polygal
**1**	0.49	0.54	0.45	0.50	0.50	0.51
**2**	0.45	0.50	0.51	0.51	0.51	0.50
**3**	0.56	0.50	.055	0.50	0.54	0.52

Table S2 Primer sequences for transcriptional analysis and comparison by qPCR and cloning for protein expression.

Primer Name	Sequence
**CA_C16SaRTsense**	GTGGGGAGCAAACAGGATTA
**CA_C 16SaRTantisense**	TGTTAACTGCGGCACAGAAG
**CA_C 0359RTsense**	GGAAGAGCTATGGGCTGGTA
**CA_C 0359RTantisense**	TTTGCTGGCATTATCCTGAA
**CA_C 1343RTsense**	CCGGGTTCAATAAATGAAGG
**CA_C 1343RTantisense**	CTGTTTCTGCCTCTCCGTCT
**CA_C359NdeIF**	ATACTACATATGCAAAAATATTCTAAA
**CA_C359XhoR**	CTAATACTCGAGAAGTGTTTCGTATTC

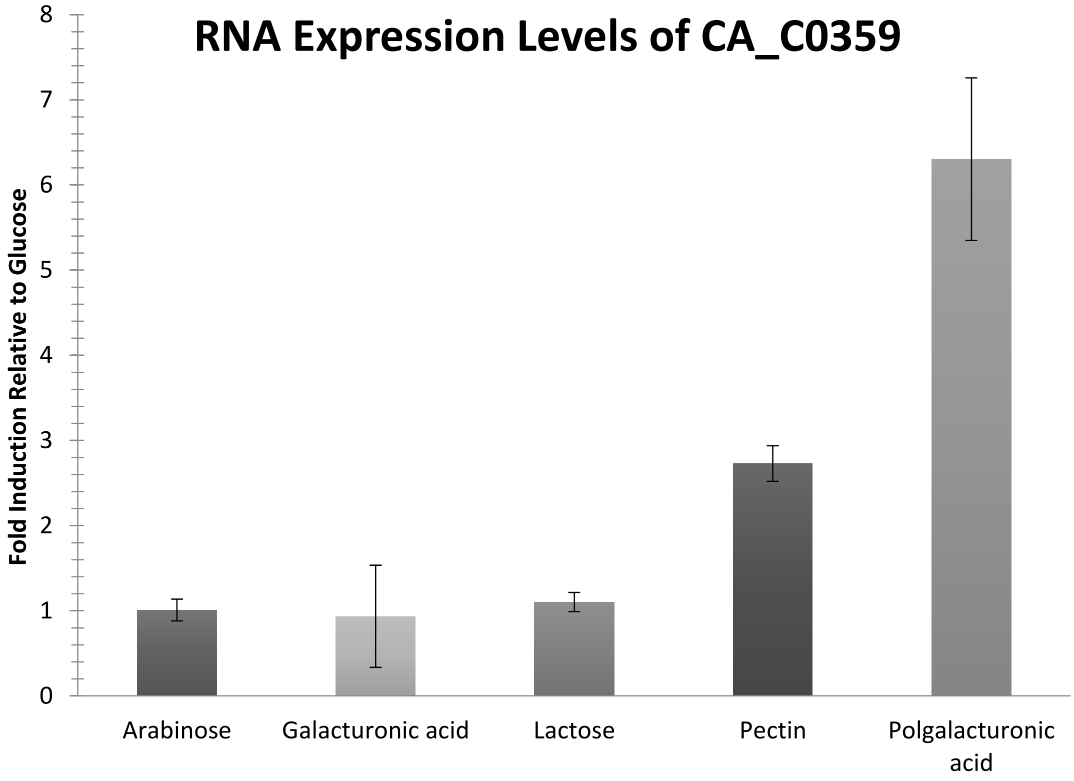

Figure S1 Transcriptional analysis by qPCR of genes induce during growth on different substrates. The fold induction is normalized to gene expression during growth on 0.5% glucose. Error bars represent plus and minus one standard deviation.

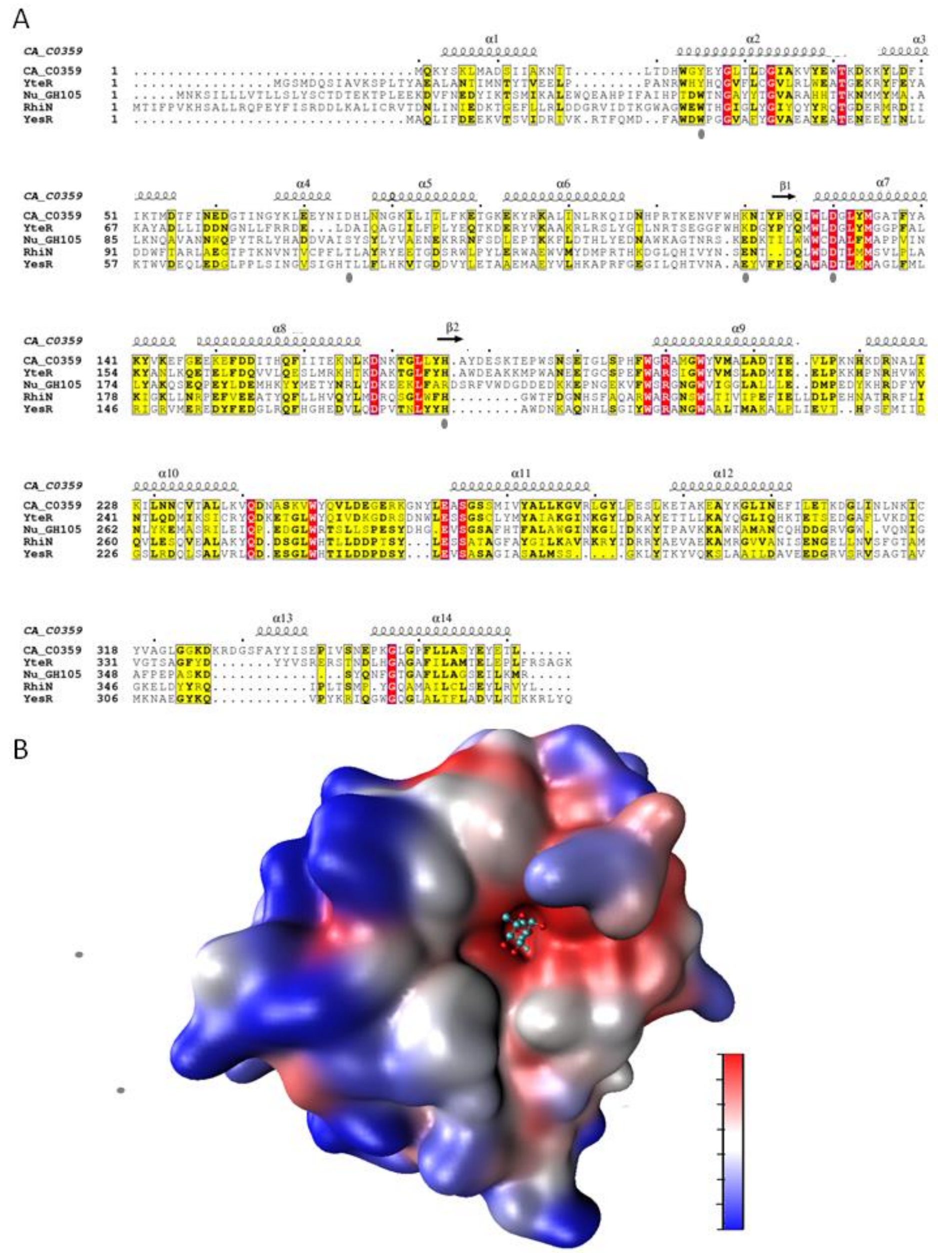

Figure S2 Conserved residues from a multiple amino acid sequence alignment of GH105 members concentrate in the putative active site of CA_C0359 structure. A. Multiple amino acid sequence alignment of proteins from CAZy family GH105 (Robert & Gouet, 2014). Highly conserved residues are highlighted red in the sequence alignment, and closely conserved residues are yellow. The location of the CA_C0359 helices and beta sheets are indicated above the alignment. Residues believed to be involved in catalytic activity for CA_C0359 contain a grey oval below the sequence B. The sequence alignment was mapped to a surface rendering of CA_C0359 crystal structure. Red indicates highly conserved residues, and blue illustrates low residue conservation. A. ΔGalA-Rha sugar is computationally modeled into the predicted active site to show the pocket appears to be conserved in the GH105 family.

Table S3 Crystallographic data collection and refinement statistics for CA_C0359(*Cac*_GH105) native crystal.

A. Data collection	
Space group	$P2_12_12_1$
a, b, c (Å)	53.3, 93.6, 156.7
Wavelength (Å)	1.075
Limiting resolution (Å) (last shell)	19.9-1.60 (1.62-1.60)
Unique reflections	103657 (10151)
R_{merge} (%)	8.6 (61.1)
Multiplicity of Observations	5.7(3.2)
Completeness overall (%)	99.8 (98.8)
$< I/\sigma(I)>$	29.3(4.5)
B.Refinement	
Resolution range (Å)	19.93-1.60 (1.66-1.6)
R-factor (%)	13.7
R-free (%)	16.3
Non-hydrogen atoms	5872
Water molecules	835
RMSD bonds(Å)	0.009
RMSD angles (°)	1.2
C. Average B factor (Å^2)	
All atoms	20.4
Water molecules	32.8
D.Ramachandran Plot	
Preferred (%)	99.2
Allowed (%)	0.8
Outliers (%)	0

Data for highest resolution shells are in parentheses.

$R_{merge}=\sum|I_i-\langle I\rangle|/\sum I_i \text{ X } 100$

$\text{R-Factor}=\sum|F_o-F_c|/\sum|F_o| \text{ x } 100$

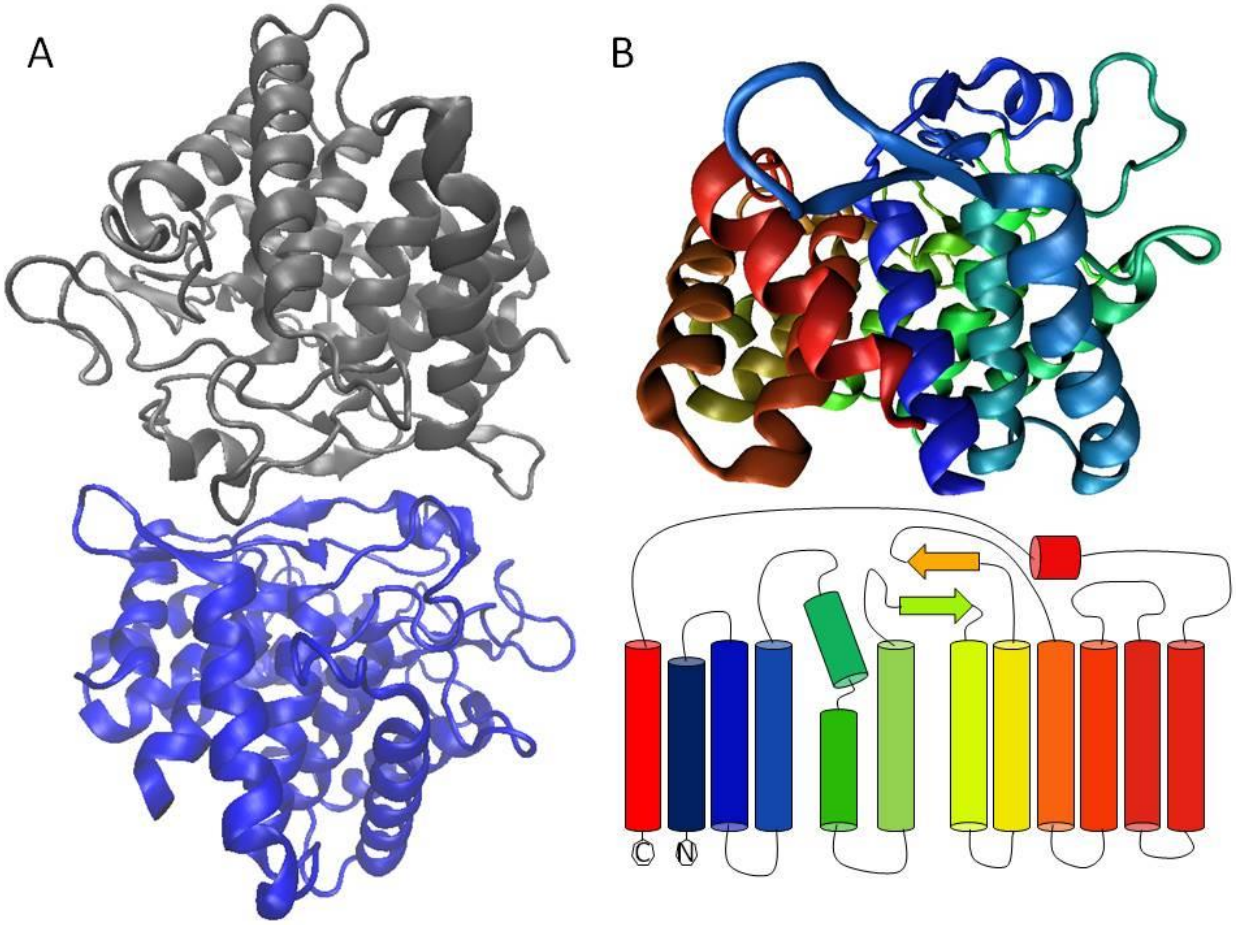

Figure S3 Crystal structure of CA_C0359 protein has two proteins in the asymetric unit and a mostly helical tertiary structure. A. The ribbon diagram of the two CA_C0359 proteins in the asymmetric unit are colored blue and grey. B. A rainbow color coded ribbon diagram of CA_C0359 with topology map below. The helices are represented as cylinders and beta sheets are represented as arrows in the topology map. The protein contains 6 α-hairpins with a 2 stranded beta sheet on top of the protein next to the putative active site.

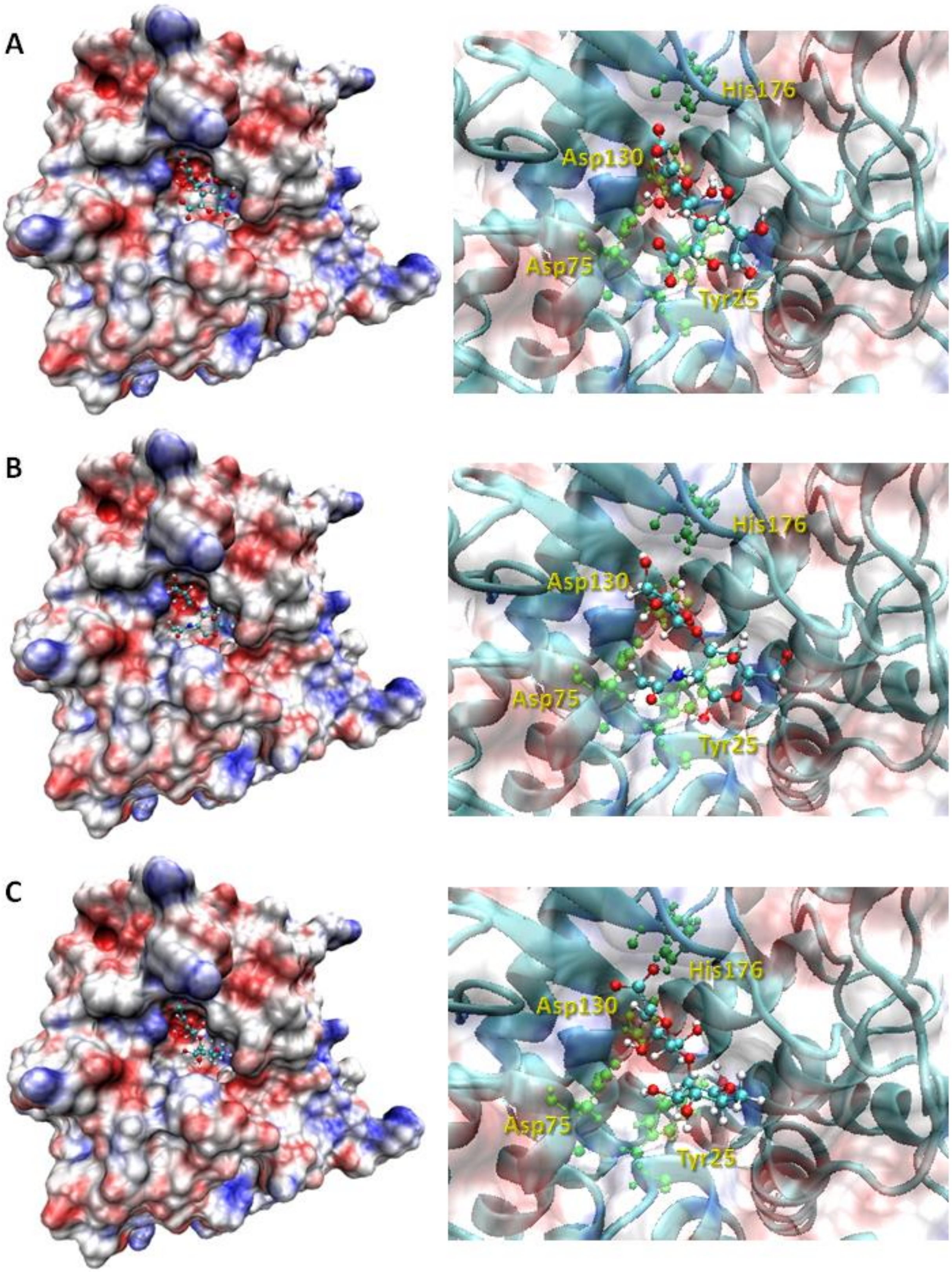
A
His176
Asp130
Asp75
Tyr25
B
His176
Asp130
Asp75
Tyr25
C
His176
Asp130
Asp75
Tyr25

Figure S4 A comparison of computationally determined binding models of A. ΔGalA-GalA, B. ΔGlcA-GalNAc, and C. ΔGalA-Rha bound to the electrostatic potential surface rendering of the CA_C0359 structure. The images to the right show the active site residues Tyr25, Asp75, Asp130, and His 176 (green, labeled in yellow) in relation to the sugars bound to the structure. CA_C0359 structure is indicated as a cyan ribbon diagram in the images to the right. The images to the left show the computationally determined modelled sugar bound to CA_C0359 structure shown in a surface rendering of the protein electrostatic potential. The unsaturated sugar binds to the negatively charged portion of the binding pocket.

A

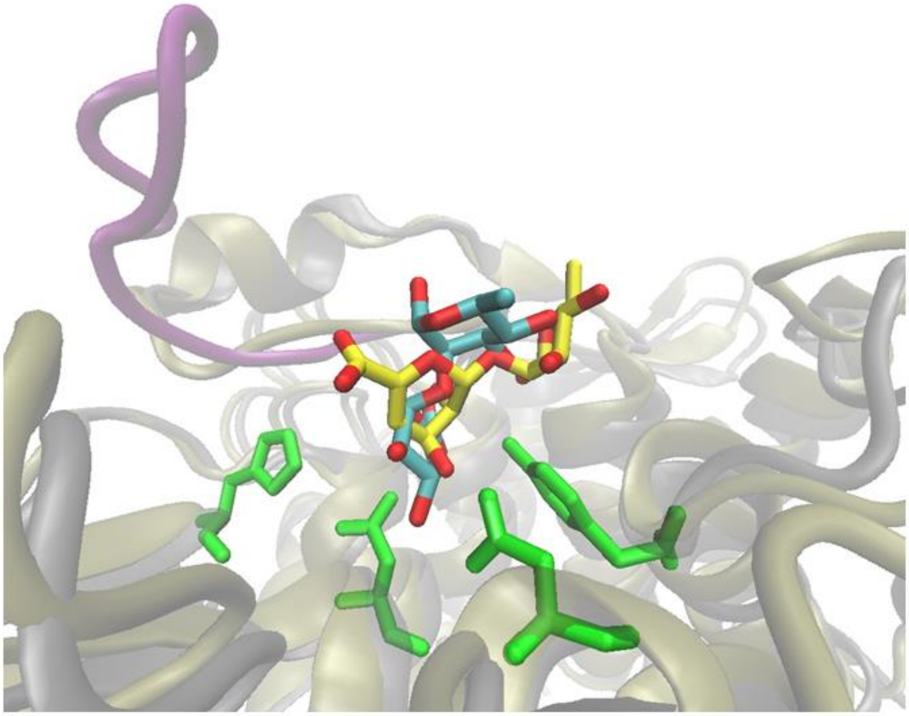

B

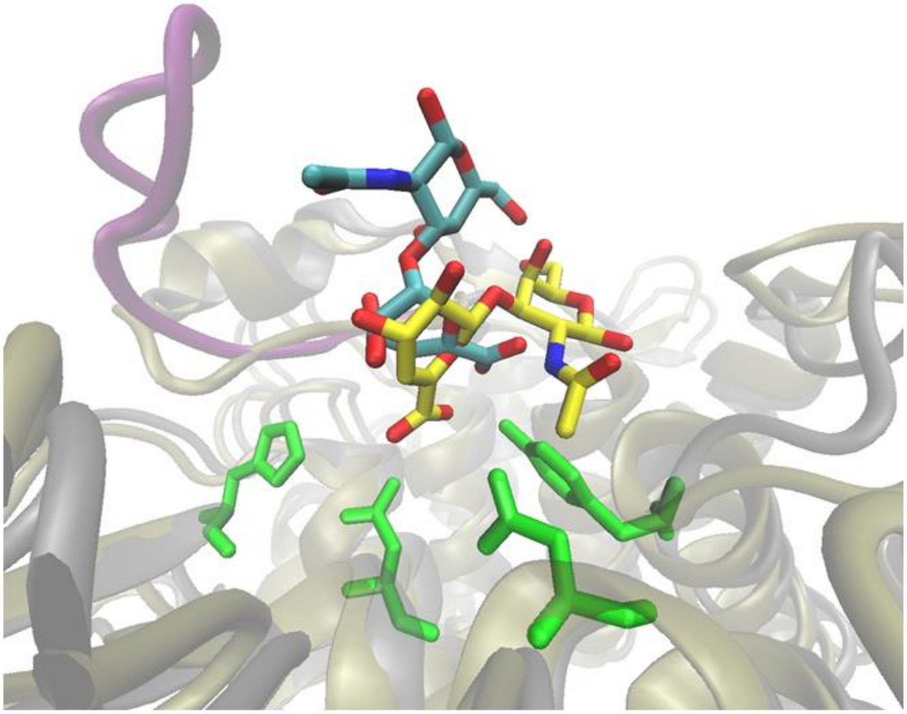

Figure S5 An overlay of computationally predicted models of sugars in the active site of CA_C0359 and structurally determined sugars from PDB submissions of YteR-2DH4 and UGL-2D8L of *B. subtilis* str 168. Panel A shows an overlay of active sites of structures CA_C0359 (grey) and YteR (mustard yellow), with the modeled sugar of ΔGalA-Rha in the active site of CA_C0359 colored yellow and the structurally determined ΔGalA-Rha in the active site of YteR colored cyan. Panel B shows an overlay of CA_C0359 (grey) and UGL (mustard yellow) with the modeled sugar of ΔGlcA-GalNAc in the active site of CA_C0359 colored yellow and the structurally determined ΔGlcA-GalNAc in the active site of UGL colored cyan. Known conserved active site residues of CA_C0359 from left to right, HIS176, Asp130, AS75 and Tyr25, are colored green. The extended protruding loop of CA_C0359 is colored magenta.
